# Predicting the Unexpected: Clinicopathological Insights into Skip Metastasis in Papillary Thyroid Carcinoma

**DOI:** 10.3390/jcm14124255

**Published:** 2025-06-15

**Authors:** Ibrahim Burak Bahcecioglu, Adile Begum Bahcecioglu, Sevket Baris Morkavuk, Yasin Hatipoglu, Sumeyra Guler, Mujdat Turan, Gokhan Giray Akgul, Nese Ersoz Gulcelik, Mehmet Ali Gulcelik

**Affiliations:** 1 Department of Surgical Oncology, Ankara Gulhane Research and Training Hospital, Health Sciences University, Ankara 06010, Turkey; dr.ibb@hotmail.com (I.B.B.); guler.sumeyra@yahoo.com (S.G.); girayakgul@hotmail.com (G.G.A.); mehmetali.gulcelik@sbu.edu.tr (M.A.G.); 2 Department of Endocrinology and Metabolism, Ankara Gulhane Research and Training Hospital, Health Sciences University, Ankara 06010, Turkey; begumbahceci@hotmail.com (A.B.B.); neseersoz.gulcelik@sbu.edu.tr (N.E.G.); 3 Department of General Surgery, Ankara Gulhane Research and Training Hospital, Health Sciences University, Ankara 06010, Turkey; yasinhatipoglu446@hotmail.com (Y.H.); mujdatturan84@hotmail.com (M.T.)

**Keywords:** papillary thyroid carcinoma, skip metastasis, lymph node metastasis

## Abstract

**Background/Objectives:** Papillary thyroid carcinoma (PTC) accounts for the majority of thyroid cancers, with lymph node metastasis, including skip metastasis (SM), playing a crucial role in guiding prognosis and therapeutic planning. SM, characterized by lateral lymph node spread in the absence of central compartment involvement, has been observed in PTC with a wide range of reported frequencies. The identification of risk factors for SM is crucial for preoperative evaluation and surgical planning. This research aims to explore the clinicopathological features and potential risk factors linked to SM in patients with PTC, while also offering valuable insights for preoperative risk evaluation. **Methods:** A retrospective cohort study was conducted on 81 PTC patients who underwent central and lateral cervical lymph node dissection (LND) in our center. Clinical, demographic, and pathological data, including age, sex, tumor size, location, subtype, extrathyroidal extension, lymphovascular invasion, and the number of lymph node metastases were analyzed. Clinicopathological characteristics were analyzed between SM-positive and SM-negative patient groups using suitable statistical methods. Additionally, a regression analysis was performed to identify the risk factors for SM. **Results:** Of the 81 patients, 17.3% (n = 14) were diagnosed with skip metastasis (SM). The SM-positive group had a significantly higher age (*p* = 0.006), smaller tumor size (*p* < 0.001), and higher rates of extrathyroidal extension (*p* = 0.006). The proportion of female patients was elevated in the SM-positive group, but this observation did not achieve statistical significance (*p* = 0.128). Tumors located in the upper pole were more common in the SM-positive group (*p* = 0.016). Multivariate analysis revealed that female sex, older age, and tumor location in the upper pole were significant risk factors for SM (*p* = 0.031, *p* = 0.004, and *p* = 0.017, respectively), while a lower number of lateral lymph node metastases was significantly associated with SM (*p* = 0.010). Additionally, an age over 43.5 years predicted SM with a sensitivity of 78.6% and a specificity of 72.7%. **Conclusions:** Skip metastasis is not uncommon in PTC and may be associated with older age, female sex, upper pole tumor location, and fewer lateral lymph node metastases. Recognizing these factors during preoperative assessment may aid in anticipating atypical lymphatic spread patterns and optimizing surgical strategies.

## 1. Introduction

Papillary thyroid carcinoma (PTC) represents the predominant form of thyroid malignancy and stands as the most common endocrine neoplasm, with its worldwide prevalence demonstrating a steady increase over recent years [1]. Cervical lymph nodes serve as the principal metastatic destination in PTC cases. Central lymph node metastasis (LNM) prevalence is documented within the 21–62% range [2], and lateral cervical LNM in thyroid malignancies occurs in approximately 20% of cases [3]. Although PTC generally has an excellent prognosis, cervical LNM can elevate the risk of locoregional recurrence and reduce the disease-free survival rate in patients with PTC [4,5]. The occurrence of lymph node metastases in PTC typically follows a predictable and sequential pattern, with PTC cells disseminating through the lymphatic system. The metastatic cascade typically originates in the central compartment, progresses to the ipsilateral lateral compartment, and finally extends to contralateral lateral or mediastinal compartments [6,7]. Skip metastasis (SM), characterized by lateral cervical lymph node involvement in the absence of central compartment metastasis, has been observed in patients with PTC at rates ranging from 2% to 38% in previous reports [8,9,10,11,12].

In PTC, ultrasonography is essential for preoperative evaluation and determining the surgical approach. It is necessary to assess both the central and lateral compartments to decide the type of dissection required. However, in the absence of sonographically suspicious lymph nodes in the central compartment, sonographers may feel somewhat reassured about the lateral compartments. This situation, however, carries the risk of missing SM. Therefore, understanding which patients have a high probability of SM is crucial for both the sonographer performing the preoperative evaluation and the surgeon determining the surgical approach.

In several previous studies, risk factors for SM have been shown to include female sex, age over 40 years, upper tumor location, small tumor size (≤1 cm), and the presence of extrathyroidal extension [13,14,15,16]. Additionally, patients with SM tend to have fewer lateral lymph node metastases compared to those without SM [17]. Despite numerous reports, existing studies on SM in PTC often suffer from methodological heterogeneity, particularly regarding how central lymph node dissection (CLND) is defined or executed. For example, some previous studies only describe CLND based on anatomical boundaries without setting a minimum lymph node count threshold, which raises concerns about the misclassification of true skip metastasis due to insufficient central sampling. This inconsistency in surgical and pathological standards hinders the accurate assessment of SM and its risk factors. A more standardized approach—such as ensuring the removal of a minimum number of central lymph nodes—may improve diagnostic accuracy and comparability across studies.

We aimed to investigate the clinicopathological characteristics and risk factors associated with SM in PTC patients, provide individual risk assessments during preoperative evaluation, and guide clinicians and surgeons in making treatment decisions.

## 2. Material and Methods

### 2.1. Study Population and Inclusion Criteria

This retrospective single-institution cohort investigation was performed from 2020 to 2023 within the Department of Surgical Oncology at Gülhane Medical Faculty, University of Health Sciences, Ankara, Turkey. The study enrolled PTC patients who received both central and lateral cervical lymph node dissection procedures at our facility, irrespective of the location where the thyroidectomy was conducted.

All patients included in this study underwent a comprehensive preoperative evaluation, including cervical ultrasonography performed by experienced endocrinologists. Suspicious lymph node characteristics were established using ultrasonographic parameters including rounded configuration, fatty hilum absence, hyperechogenic pattern, microcalcification detection, cystic transformation, and peripheral blood flow. Upon identifying these suspicious attributes in lateral cervical lymph nodes, an ultrasound-guided fine-needle aspiration biopsy (FNAB) was executed. When cytological results remained inconclusive or required additional validation, thyroglobulin levels were assessed in aspirate washout samples (FNA-Tg). Only patients with cytologically confirmed metastasis or those meeting both biochemical criteria—FNA-Tg levels exceeding 10 ng/mL and an FNA-Tg to serum Tg ratio greater than 1—were considered to have lateral lymph node metastasis and included in this study.

As skip metastasis, by definition, requires evaluation of both central and lateral compartments, patient selection was restricted to those who experienced both central and lateral neck dissection procedures. This ensured a consistent surgical approach and enabled accurate assessment of skip metastasis in accordance with the study objectives.

Nodal dissection techniques adhered to recognized anatomical reference points. Central compartment dissection (CLND) addressed level VI, with boundaries established by the hyoid bone cephalad, sternal notch caudad, and carotid vessels laterally. Lateral compartment dissection (LND) incorporated levels II–V, extending cephalocaudally from the skull base to clavicle, and mediolaterally from the sternohyoid muscle’s lateral edge to the trapezius muscle’s anterior aspect. All patients underwent both CLND and LND on the ipsilateral side of the tumor, in accordance with the preoperative evidence of lateral lymph node metastasis.

The eligibility criteria encompassed patients aged ≥16 years presenting a PTC diagnosis who underwent central and lateral cervical lymph node dissection (LND) at our institution. An adequate central LND was defined as the removal of ≥5 central lymph nodes. This threshold was chosen to ensure that central compartment involvement was thoroughly evaluated, minimizing the risk of misclassifying skip metastasis due to insufficient sampling. The exclusion criteria comprised patients who underwent either a central or lateral LND alone, those with an inadequate central LND (<5 lymph nodes removed), and cases with incomplete pathology data from a thyroidectomy performed at another center. Patients with missing or incomplete medical records regarding LND details were also excluded.

### 2.2. Study Design

Clinical, demographic, and pathological data were extracted by retrospectively reviewing patient files. Variables such as age, sex, PTC subtype (classical, oncocytic, tall cell), tumor size, tumor location (upper pole, medial lobe, lower pole, isthmus), according to histopathological examination, the presence of extrathyroidal extension, presence of multifocality, lymphovascular invasion (LVI), type of lymph node dissection, and the number of lymph nodes were recorded. The study population was stratified into two groups: patients exhibiting lateral cervical lymph node metastasis without concurrent central lymph node metastasis (SM-positive group) and patients showing metastatic involvement of both central and lateral lymph nodes (SM-negative group).

### 2.3. Statistical Analysis

Data processing and analysis were executed through SPSS statistical software version 22.0 (SPSS Inc., Chicago, IL, USA). Numerical variables are reported as median values with their corresponding ranges, whereas nominal variables are displayed through frequency distributions and percentage calculations. Distribution patterns were examined using a Kolmogorov–Smirnov normality test enhanced with a Lilliefors adjustment. Inter-group statistical evaluations utilized independent t-testing for parametrically distributed datasets and Mann–Whitney U testing for non-parametric data distributions. Logistic regression modeling with binary outcomes was employed to determine the contributing factors associated with skip metastasis development. Variables yielding *p*-values below 0.25 during the initial univariate screening were subsequently incorporated into the comprehensive multivariate analytical framework. The occurrence of skip metastasis was established as the primary outcome measure. Explanatory variables encompassed sex, age, tumor size, tumor location, multifocality, extrathyroidal extension, lymphovascular invasion, and number of lateral lymph node metastases, all evaluated for their predictive influence on skip metastasis manifestation. A receiver operating characteristic curve methodology was implemented to determine age-related diagnostic precision in skip metastasis detection. Multiple hypothesis testing was controlled through Bonferroni correction procedures to minimize false discovery rates. The criterion for statistical significance was established at *p*-values less than 0.05.

## 3. Results

### 3.1. Baseline Sample Characteristics

Between 2020 and 2023, a total of 151 patients with papillary thyroid carcinoma (PTC) underwent a neck dissection at our department. Of these, 49 patients were excluded due to central or lateral dissections being performed at another center. Additionally, 16 patients were excluded due to incomplete pathology reports from a thyroidectomy performed at another center, 3 patients due to an insufficient number of central lymph nodes dissected, and 2 patients for being under 16 years of age. As a result, a total of 81 patients fulfilling the inclusion criteria were enrolled in the final analysis.

The study encompassed 81 patients who underwent retrospective evaluation. Female patients comprised 49 cases (60.5%) of the cohort. Patient age averaged 41.9 ± 15 years (spanning 16–75 years), with male patients demonstrating a mean age of 43.2 ± 17 years (range: 19–74) and female patients showing an average of 41.1 ± 13.6 years (range: 16–75). Fifteen patients had a history of thyroidectomy, while 66 underwent primary surgical treatment. Skip metastasis was detected in 17.3% (n = 14) of the patients. Table 1 displays the demographic and histopathological profiles of the study cohort.

### 3.2. Clinicopathological Parameter Assessment: Skip Metastasis-Positive vs. Skip Metastasis-Negative Cohorts

The SM-positive cohort exhibited a significantly greater age than the SM-negative cohort (*p* = 0.006). Conversely, tumor size was markedly diminished in SM-positive patients (*p* < 0.001). Moreover, these patients exhibited fewer metastatic lateral lymph nodes than their SM-negative counterparts (*p* = 0.013). Tumor location also differed significantly between groups: half of the tumors in the SM-positive group were situated in the upper pole, while in the SM-negative group, the majority were found in the middle lobe (*p* = 0.016). The distribution of skip metastasis across T stages was evaluated; although SM was more frequently observed in T1a and T1b tumors, while none of the patients with T3 tumors had SM, this association did not reach statistical significance (*p* = 0.109). Between-group comparisons revealed no statistically meaningful differences for maximum metastatic lymph node diameter, histopathological subtype, extrathyroidal extension, multifocality, or lymphovascular invasion between SM-positive and SM-negative patients (*p* > 0.05). The association between skip metastasis and clinicopathological parameters is summarized in Table 2.

### 3.3. Risk Factor Analysis for Skip Metastasis Using Regression Modeling

Skip metastasis predictors were examined and documented in Table 3 and Table 4.

In the univariate regression analysis, older age (*p* = 0.013), smaller tumor size (*p* = 0.014), upper pole tumor location (*p* = 0.006), and lower metastatic lateral lymph node count (*p* = 0.032) showed significant associations with skip metastasis. Multivariate regression analysis identified female sex (*p* = 0.031), older age (*p* = 0.004), upper pole tumor location (*p* = 0.017), and lower metastatic lateral lymph node count (*p* = 0.010) as independent predictors of skip metastasis.

### 3.4. Optimal Cut-Off Determination for Age and Tumor Size in Skip Metastasis: ROC Analysis

**Figure 1:**
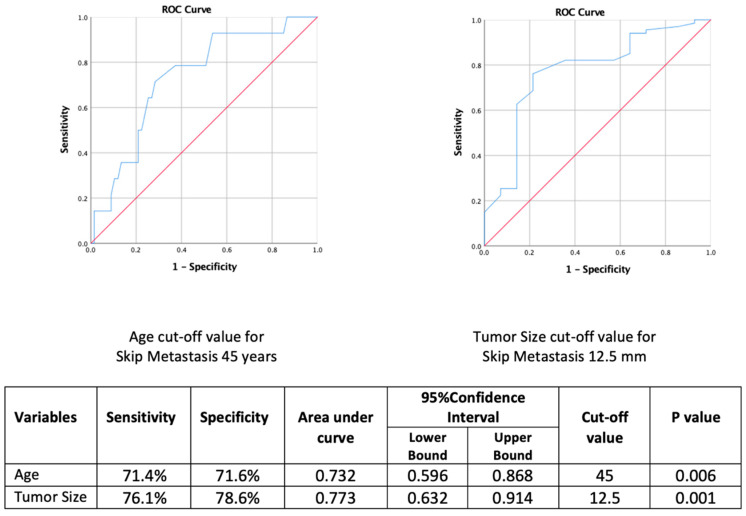
Predictive Value of Age and Tumor Size for Skip Metastasis: ROC Curve Analysis.

An age threshold above 45 years was found to predict skip metastasis with 71.4% sensitivity and 71.6% specificity. Similarly, a tumor diameter less than 12.5 mm demonstrated 76.1% sensitivity and 78.6% specificity in identifying patients with skip metastasis (Figure 1).

## 4. Discussion

This research aimed to characterize the clinical and pathological features of patients with papillary thyroid carcinoma (PTC) who were diagnosed with skip metastasis and explore the associated predictive factors. Our results indicate that SM occurred in 17.3% of patients, consistent with previously reported incidence rates ranging from 2% to 38% in the literature [8,9,10,11,12,18]. We found that older age, female sex, upper pole tumor location, and fewer lateral lymph node metastases were significantly associated with SM, in agreement with several prior studies [13,14,15,16,17].

We identified a cut-off value of 45 years, with acceptable sensitivity and specificity (71.4% and 71.6%, respectively), which is consistent with the findings of Zhao et al., who also reported an age over 45 years as a risk factor for SM in their systematic review and meta-analysis [14]. Wang et al. also identified age as a key factor in their predictive nomogram model for SM, supporting the relevance of age in preoperative risk stratification [15]. Although female sex was not significantly different between SM-positive and SM-negative groups in the univariate analysis (*p* = 0.128), it emerged as an independent predictor in multivariate regression, consistent with some studies and large meta-analyses [14,16,19], though others have reported conflicting results [13,15]. This suggests that the role of sex may be context-dependent and could be influenced by hormonal, anatomical, or detection biases.

In our study, tumor size showed a significant association with SM in the univariate analysis; however, this association disappeared after multivariate adjustment. This suggests that the effect of tumor size on SM may be mediated or confounded by other variables, particularly tumor location and the extent of lymph node involvement. Notably, tumor location in the upper pole was significantly associated with SM in both univariate and multivariate analyses in our cohort, demonstrating the strongest association among all independent predictors; this finding supports the hypothesis that upper pole tumors may drain directly to the lateral neck nodes via the superior thyroidal lymphatic pathway, thereby bypassing the central compartment. Indeed, previous studies have consistently shown that smaller tumors, especially those located in the upper pole, are more likely to bypass the central lymph nodes and metastasize directly to the lateral compartment [20]. For instance, Wang et al. identified a tumor size ≤1 cm and upper pole location as independent predictors of SM, with tumor location in the upper portion showing a particularly strong association (OR = 6.799, *p* < 0.001) [15]. Similarly, in a systematic review and meta-analysis, Zhao et al. reported that both a tumor diameter ≤10 mm and upper tumor location were significantly associated with SM, with odds ratios of 2.23 and 3.60, respectively [14]. These findings support the notion that tumor location may play a more dominant role than tumor size alone in the pathogenesis of SM, potentially explaining why tumor size lost its statistical significance in our adjusted model.

We also observed that patients with SM had fewer metastatic lateral lymph nodes compared to those without SM, suggesting a more limited pattern of lateral nodal involvement. This observation aligns with Lei et al.’s findings, demonstrating that skip metastasis patients exhibited not only reduced lateral lymph node metastases but also an increased likelihood of single-level involvement, primarily affecting Level II nodes (*p* < 0.05) [17]. These results support the hypothesis that SM may reflect a distinct pattern of lymphatic spread—possibly due to direct drainage from the upper pole via the superior vascular pedicle—resulting in more localized lateral involvement and fewer metastatic nodes. This might indicate a less extensive lymphatic spread in SM cases, or alternatively, a distinct biological behavior that differentiates SM from conventional lymphatic dissemination patterns.

In our study, neither extrathyroidal extension (ETE) nor PTC subtype was significantly associated with skip metastasis. This finding aligns with the results of the systematic review and meta-analysis by Zhao et al., which included 28 studies and over 10,000 patients, that reported that ETE was not significantly related to skip lateral lymph node metastasis (OR = 1.07, 95% CI = 0.83–1.39, *p* = 0.598) [14]. Similarly, other tumor characteristics such as multifocality, capsular invasion, and histological subtype were also not found to be predictive of SM in that analysis. In contrast, a study by Nie et al. suggested that ETE was significantly associated with lateral lymph node metastasis in general but did not establish a direct link with skip metastasis specifically [18]. These discrepancies may reflect differences in patient populations, inclusion criteria, or methodological definitions of ETE and SM. Our findings further support the notion that SM may follow a distinct biological or anatomical pathway, independent of classical markers of aggressive disease such as ETE or unfavorable tumor subtypes.

Unlike many previous studies, our cohort was strictly limited to patients with an adequate central lymph node dissection, defined as the removal of five or more central lymph nodes. This methodological criterion was intentionally applied to minimize the misclassification of skip metastasis due to undetected microscopic involvement in an insufficiently dissected central compartment. In several influential studies, such as the nomogram-based prediction model by Wang et al. [15] and the external validation study by Li et al. [16], central lymph node dissections were described anatomically (e.g., from the hyoid bone to the sternal notch) without specifying the number of dissected nodes. Although these descriptions suggest standard anatomical boundaries, they lack an objective numerical threshold, which may risk underestimating central involvement. By contrast, our strict inclusion criterion provides a quantifiable and reproducible definition of an adequate central dissection. This distinction enhances the internal validity of our findings and strengthens the accuracy of skip metastasis classification within our cohort.

The identification of reliable predictors for skip metastasis holds critical implications for surgical planning and preoperative lymph node evaluation in papillary thyroid carcinoma. Our findings support the incorporation of variables such as age, sex, tumor location, and the number of metastatic lymph nodes into clinical risk assessment algorithms. Future prospective, multicenter studies with standardized definitions—particularly regarding the extent of central lymph node dissections—are necessary to validate and refine the predictive models. Additionally, integrating imaging criteria and molecular markers (e.g., BRAF, TERT mutations) with clinicopathological factors may enhance the precision of SM prediction. From a clinical standpoint, tailoring the extent of lymph node dissections based on individualized SM risk could reduce surgical morbidity while preserving oncologic efficacy. Ultimately, these efforts may contribute to more personalized and efficient management strategies for patients with PTC.

This study has several limitations. First, it was conducted retrospectively at a single tertiary center with a relatively small sample size, which may limit the generalizability of the findings. Additionally, due to the retrospective design, the possibility of unmeasured confounding variables cannot be completely excluded. Molecular and genetic markers, which may influence metastatic patterns, were not evaluated in this study. Nonetheless, this study has several strengths. Our institution serves as a referral center for thyroid cancer patients with cervical metastasis, and the surgical oncology team has extensive experience in the management of these cases. All patients underwent standardized preoperative neck mapping with ultrasonography, interpreted using uniform diagnostic criteria across cases. Moreover, we applied a strict inclusion criterion of at least five dissected central lymph nodes, which minimizes the likelihood of false-positive skip metastasis diagnoses due to undetected central node involvement. These methodological strengths enhance the internal validity and reliability of our findings.

## 5. Conclusions

Skip metastasis represents a distinct pattern of lymphatic spread in papillary thyroid carcinoma. Our study identified older age, female sex, upper pole tumor location, and a lower number of lateral lymph node metastases as factors significantly associated with SM. These findings highlight the importance of carefully assessing these variables during preoperative ultrasonography and surgical planning, even in the absence of central lymph node involvement. Incorporating these predictors into clinical decision-making may contribute to more accurate risk stratification and improved disease management. To confirm these findings and enhance preoperative risk assessment models, future research should focus on larger, multicenter prospective cohorts.

## Figures and Tables

**Table 1:** Patient Demographics and Histopathological Features.

**Age, Year, Mean ± SD**	41.9 ± 15 (16–75)
**Sex: n (%)**	
Female	49 (60.5%)
**Primary/Recurrent Cases: n (%)**	
Primary	66 (81.5%)
Secondary	15 (18.5%)
**Tumor Location(Longitudinal): n (%)**	
Upper Pole	18 (22.2%)
Middle Lobe	46 (56.8%)
İnferior Pole	9 (11.1%)
Isthmus	8 (9.9%)
**Tumor Location (axial): n (%)**	
Right lobe	43 (53.1%)
Left Lobe	30 (37%)
Istmus	8 (9.9%)
**Tumor Subtypes: n (%)**	
Classic Papillary	69 (85.2%)
Tall Cell Papillary	6 (7.4%)
Diffuse sclerosing	6 (7.4%)
**Tumor Size, mm, mean ± SD**	20 ± 12.6 (9–60)
**Extrathyroidal Extension: n (%)**	22 (27.2%)
**Multifocality: n (%)**	39 (48.1%)
**LVI: n (%)**	60 (74.1%)
**Total Lymph node dissection, number, mean ± SD**	32.8 ± 15.6 (13–103)
Central	10.8 ± 6.0 (5–29)
Lateral	21.8 ± 13.2 (6–98)
**Number of Lymph Node Metastases, mean ± SD**	8.9 ± 6.8 (1–31)
Central ratio	4.5 ± 4.5 (0–22)
Lateral ratio	4.4 ± 3.5 (1–18)
**Maximal diameter of Lymph node metastasis, mm, mean ± SD**	15.7 ± 10.3 (5–60)
**Skip Metastasis: n (%)**	14 (17.3%)

SD, standard deviation; Min, minimum; Max, maximum; LVI, lymphovascular invasion.

**Table 2:** Association between Skip Metastasis and Clinicopathological Parameters.

Clinicopathological Parameters	No. of Patients (%) SM-Negative Group SM-Positive Group (67 Patients 82.7%) (14 Patients 17.3%)		*p* Value
**Age, year, median, min-max**	38 (16–75)	50 (24–74)	*p* = 0.006 ^U^
**Sex: n (%)**			
Female	38 (56.7%)	15 (78.6%)	*p* = 0.128 ^x2^
**Tumor Size, mm, mean ± SD**	19 (3–60)	11 (0–35)	*p* < 0.001 ^U^
**Maximal diameter of lymph node metastasis, mm, median, min-max**	12 (5–60)	12.5 (6–32)	*p* = 0.698 ^U^
**Number of Lateral lymph node metastases**	4 (1–18)	2 (1–7)	*p* = 0.013 ^U^
**Primary/Secondary Cases: n (%)**			*p* = 0.228 ^x2^
Primary	53 (79.1%)	13 (92.9%)	
Secondary	14 (20.9%)	1 (7.1%)	
**Tumor Location: n (%)**			*p* = 0.016 ^x2^
Upper Pole	12 (17.9%)	7 (50%)	
Middle Lobe	42 (62.7%)	3 (21.4%)	
İnferior Pole	6 (9%)	3 (21.4%)	
isthmus	7 (10.4%)	1 (7.1%)	
**Tumor Subtypes: n (%)**			*p* = 0.230 ^x2^
Classic Papillary	55 (82.1%)	14 (100%)	
Tall Cell Papillary	6 (9%)	0	
Diffuse Sclerosing	6 (9%)	0	
**Extrathyroidal Extension: n (%)**	20 (29.9%)	2 (14.3%)	*p* = 0.234 ^x2^
**Multifocal Status: n (%)**	33 (49.3%)	6 (42.9%)	*p* = 0.663 ^x2^
**LVI Status: n (%)**			
Present	49 (73.1%)	11 (78.6%)	*p* = 1 Fischer’s exact
**T stage**			
**T1a**	12 (66.7%)	6 (33.3%)	
**T1b**	30 (83.3%)	6 (16.7%)	*p* = 0.109 ^x2^
**T2**	12 (85.7%)	2 (14.3%)	
**T3**	13 (100%)	0 (0%)	

SM, skip metastasis; Min, minimum; Max, maximum; X2, χ2 tests; U, Mann–Whitney U test.

**Table 3:** Univariate Regression Analysis of Clinicopathological Factors in Skip Metastasis.

Dependent Variable	B	OR	95% Cl for OR	*p* Value
**Sex**	1.029	2.798	0.715–10.957	0.140
Female				
*(Reference variable male)*				
**Age**	0.051	1.053	1.011–1.097	0.013
**Tumor Size**	−0.105	0.900	0.829–0.979	0.014
**Lateral lymph node metastasis, number**	−0.357	0.700	0.505–0.970	0.032
**Tumor Location**				
Upper Pole	1.407	4.083	1.412–40.455	0.006
Middle Lobe	−0.693	0.500	0.045–5.514	0.856
Inferior Lobe	1.253	3.500	0.284–43.161	0.229
*(Reference variable istmus)*				
**Tumor Subtypes**				N/A
Classic				
Tall cell				
Diffuse sclerosing				
**Extrathyroidal Extension**	0.937	2.553	0.523–12.467	0.247
Present				
*(Reference variable absent)*				

OR, odds ratio; CI, confidence interval.

**Table 4:** Multivariate Regression Analysis of Clinicopathological Factors in Skip Metastasis.

Dependent Variable	B	OR	95% Cl for B	*p* Value
**Sex**	3.572	35.589	1.390–910.943	0.031
Female				
*(Reference variable male)*				
**Age**	0.138	1.148	1.044–1.261	0.004
**Tumor Size**	−0.118	0.888	0.767–1.029	0.113
**Lateral lymph node metastasis, number**	−7.33	0.480	0.275–0.838	0.010
**Tumor Location**				
Upper Pole	4.492	89.295	2.269–3513.496	0.017
Middle Lobe	−0.969	0.380	0.016–8.784	0.546
Inferior Lobe	4.073	58.761	1544–2236.151	0.028
*(Reference variable istmus)*				
**Extrathyroidal Extension**	0.990	2.692	0.148–49.102	0.504
Present				
*(Reference variable absent)*				

OR, odds ratio; CI, confidence interval.

## Data Availability

The research datasets utilized in this investigation can be obtained for academic purposes from the corresponding author upon reasonable request.

## Abbreviations

PTC	Papillary thyroid carcinoma
SM	Skip metastasis
LND	Lymph node dissection
LNM	Lymph node metastasis
LVI	Lymphovascular invasion
ETE	Extrathyroidal extension
